# SNPs Array Karyotyping in Non-Hodgkin Lymphoma

**DOI:** 10.3390/microarrays4040551

**Published:** 2015-11-12

**Authors:** Maryam Etebari, Mohsen Navari, Pier Paolo Piccaluga

**Affiliations:** Department of Experimental, Diagnostic, and Specialty Medicine; Bologna University School of Medicine; Hematopathology Unit, S. Orsola-Malpighi Hospital, Bologna 40138, Italy; E-Mails: maryam.etebari@gmail.com (M.E.); mohsen.navari@gmail.com (M.N.)

**Keywords:** non-Hodgkin lymphoma, single nucleotide polymorphism (SNP) array, genetic aberrations

## Abstract

The traditional methods for detection of chromosomal aberrations, which included cytogenetic or gene candidate solutions, suffered from low sensitivity or the need for previous knowledge of the target regions of the genome. With the advent of single nucleotide polymorphism (SNP) arrays, genome screening at global level in order to find chromosomal aberrations like copy number variants, DNA amplifications, deletions, and also loss of heterozygosity became feasible. In this review, we present an update of the knowledge, gained by SNPs arrays, of the genomic complexity of the most important subtypes of non-Hodgkin lymphomas.

## 1. Introduction

Non-Hodgkin lymphomas (NHL) are a heterogeneous group of malignancies that originate from the lymphatic system and, in particular, from either immature or mature/peripheral lymphocytes [1]. Their classification is primarily based on the cell of origin (*i.e.*, B or T cell) or histogenesis, which is further narrowed into different categories based on morphology, phenotype, genetic and clinical characteristics. Therefore, the diverse types of NHL significantly vary in both their clinical features, being defined as either indolent or aggressive, and pathobiology. As far as the latter is concerned, in the last decade tremendous advances have been made in the understanding of the molecular pathogenesis of lymphomas leading in some instances to a real molecular classification, as in the case of diffuse large B-cell lymphomas (DLBCL) (see below) [2].

Genetic aberrations, such as copy number variants (CNVs) and translocations have been shown to be specifically correlated with certain human cancers and, quite often, with hematological malignancies including lymphomas. In this regard, various technological advances in molecular analyses have greatly enhanced our ability to identify specific genetic aberrations in any tumor type [3]. In particular, the introduction of high-throughput technologies such as microarrays allowed fast and relatively cheap extensive studies leading to the discovery of several genetic aberrations [3]. Importantly, their identification has a practical impact, having an important role in diagnosis, prognosis, and subsequent choice of therapy in cancers [4,5,6,7,8].

Traditionally, genomic lesions in cancer specimens have been recognized using metaphase cytogenetic (MC), fluorescent *in situ* hybridization (FISH) and Sanger sequencing. However, though still relevant, these methods impose several limitations like the dependence on the availability of dividing cells and resolution restrictions (MC) [9]. Albeit the abilities of FISH and sequencing in triumphing over some of the above mentioned disadvantages like the lack of dependency upon dividing cells, they are limited in terms of being applicable to the candidate regions, making them unsuitable for the genome-wide screenings [10].

These drawbacks were further overcome with the introduction of high resolution methods such as comparative genomic hybridization (CGH) and SNP microarrays, enabling us with genome-wide molecular karyotyping possibilities. Besides the lack of need for dividing cells, above all SNP arrays provide us with the opportunity of the detection of several types of genetic lesions, by progressively expanding the resolution of DNA analysis (explained below) [11,12,13,14,15,16].

Here, we highlighted the most recent findings obtained from global analysis of genetic aberrations in some of the most common subtypes of NHL of B- and T-cell origin, including DLBCL, follicular lymphoma (FL), mantel cell lymphoma (MCL), marginal zone B cell lymphoma (MZL), and peripheral T cell lymphomas (PTCLs).

## 2. Single Nucleotide Polymorphism (SNP) Array and Its Applications

Single nucleotide polymorphisms (SNPs) are defined as variations of a DNA sequence at single nucleotide level which are found in a high proportion of human genome (*i.e.*, more than 1%). SNPs might occur in any position in the genome. These polymorphisms have a wide effect on human health, ranging from disease development to response to the therapies [17]. Thus, technologies like SNP arrays were recently applied in order to screen the genome at global level allowing to better define correlations between genetic patterns and phenotypes, including diseases [17,18].

Several different companies offer the related technology. However, as far as the experience in human lymphomas is concerned, two of them (*i.e.*, Affymetrix, Santa Clara, CA, USA and Illumina, San Diego, CA, USA) demonstrated their leadership, offering arrays characterized by high reliability and reproducibility. Of note, the two technologies, albeit being based on different chemistries, show some common features [19]. Basically, a microarray/chip harboring unique probes for each SNP is prepared. The target DNA is then fragmented, labeled and hybridized to the probes present on the chip. A potential binding between the target DNA and the SNP probe is evaluated based on the intensity of the signal, which could provide the researcher with the information about the copy number state and the genotype [20,21,22,23]. As a result, genetic imbalances such as copy number variants (CNVs) and loss or gain of heterozygosity (LOH and GOH, respectively) can easily be detected [24,25,26,27,28,29]. Particularly, besides resolution, the latter cannot be identified by MC. In fact, LOH is generally caused by monoallelic deletions, the lost segment being then replaced by the same region of its homologous chromosome, resulting in a so called copy neutral loss of heterozygosity (CN-LOH) or uniparental disomy (UPD) [18,30,31,32]. The acquired homozygosity resulting from UPD could lead to development of tumors, as largely indicated by SNPs array-based studies [33,34].

## 3. SNP Array Analysis in Non-Hodgkin Lymphoma

### 3.1. Diffuse Large B Cell Lymphoma

Diffuse large B-cell lymphoma (DLBCL) is the most frequent NHL subtype, accounting for about 30% of all lymphoma cases worldwide. Although overall presenting as a unique entity, with the usage of gene expression profiling, it turned out that DLBCL is constituted by at least two distinct subtypes: germinal center B-cell like (GCB-DLBCL) and activated B-cell like (ABC-DLBCL) [2]. Furthermore, a third group of DLBCL cases exists, which have been defined as “gray zone”, which are not classifiable as either ABD or GCB subtypes [35].

In 2003, Wessendorf *et al*. [36] first demonstrated that increasing sensitivity of cytogenetic analysis by matrix comparative genomic hybridization (M-CGH) could allow to detect previously uncovered lesions in DLBCL and other aggressive lymphoma specimens. Among others, the authors identified small amplifications affecting genes involved in lymphoma pathogenesis such as *BCL2*, *REL*, *CCND1*, *CCND2*, *JAK2*, *FGF4* and *MDM2*. This basic evidence prompted further research toward more and more sensitive tools. Since then, in fact, several studies have been carried out using SNP arrays alone or in combination with other methods to uncover unidentified genes involved in DLBCL (Table 1) [37,38,39,40,41,42,43,44,45,46,47,48]. While some features, e.g., gains on chromosome 7, are common between the two subtypes, many others such as gains on chromosomes 1, 7, 12, 3, 18, 2, 19 and 13 and losses on chromosomes 9, 6, 7, 15 and 17 might affect the differences between the two subtypes. Among the affected genes by gains, *REL*, *BCL2*, *BCL11A* and mir-17, and among the affected genes by losses *PTEN*, *PRDM1*, *TP53*, *TNFAIP3*, *JAK2*, *BACH2*, *CASP8AP2*, *HDAC7A* and *FAS* are worth to mention [37,38,39,40,41,42,43,44,45,46,47,48].

As far as the differences between the two subtypes of DLBCL are concerned, Scholtysi *et al.* [43] conducted a study mainly focused on the matter, analyzing 148 primary tumors (including 79 GCB-DLBCL, 49 ABC-DLBCL and 20 unclassified cases) [42,43]. Collectively, they found 24 and 38 regions of recurrent gains and losses and 38 regions of recurrent genomic losses, respectively, averaging 25 and 19 imbalances per case for ABC-DLBCL and GCB-DLBCL, respectively. Among them, a recurrent deletion was found in 19p13.3 in several primary cases, which included two members of Tumor Necrosis Factor superfamily, namely *TNFSF7* and *TNFSF9*. Furthermore, they identified several copy number variations with substantially differential frequencies among the two subtypes of DLBCL. For example, a loss on chromosome 9 (a region covering tumor suppressor genes *CDKN2A* and *CDKN2B*) was found in 46.9% of ABC-DLBCL cases, while the occurrence of this loss in GCB-DLBCLs was only 12.7%. Other examples include gain on chromosome 2 (including *REL* and *BCL11A* genes) with an occurrence rate of 10.2% and 30.4% for ABC- and GCB-DLBCL, respectively, gains of HDAC7A on chromosome 12 mainly observed in GCB-DLBCL (38% of cases as compared to 14.3% in ABC-DLBCL) and predominant losses of *BACH2* and *CASP8AP2* ABC-DLBCL (34.7% *vs.* 20.3% in GCB subtype) [42,43].

**Table 1:** The most important recurrent genetic aberrations in diffuse large B-cell lymphoma (DLBCL), as discussed in the text.

Location	Alteration	Candidate Genes	References
1q	Gain	*CD58*	[38,42,47]
2	Gain	*REL*, *BCL11A*	[37,38,40,41,42,49]
3	Gain	*FOXP1*	[42,45]
6q15	Loss	*BACH2*, *CASP8AP2*	[39,42]
6q21	Loss	*PRDM1*	[38,42]
6q23	Loss	*TNFAIP3 (A20)*, *MAP3K5*	[38,42,47]
7	Gain, Loss	-	[37,38,39]
8p	Loss	-	[41]
9p21	Loss	*CDKN2A*, *CDKN2B*	[38,47,49]
9p24	Gain	*JAK2*	[47]
9q34	Gain	*NOTCH1*	[47]
9	Loss	*INK4a/ARF*	[45]
10q	Loss	*FAS*, *PTEN*	[38,40,45,47]
10	Loss	*PTEN*	[45]
11p11	LOH	*PTPRJ*	[46]
11q25	Gain	*LOC283177*	[48]
12	Gain	*CDKN1B*	[49]
12p13	Gain	*FOXM1*	[40]
12q13	Gain	*MAP3K12*	[40]
12q	Gain	*HDAC7A*	[37,42]
13	Gain	*mir-17-92*	[45]
13q14	Gain, Loss	*RB1*	[38,49]
15	Loss	*53BP1*	[42,44]
17p	Loss	*TP53*	[37,38,40,42,49]
18q21	Gain	*BCL2*	[37,38,42,49]
19p13	Loss	*TNFSF7*, *TNFSF9*, *CD70*	[38,40,42]
19q13	Gain	*PRMT1*, *BCL2L12*	[38]

Very recently, Dias *et al.* [47] used an intercross of public datasets from three different platform types (*i.e.*, SNP array, CGH array and Gene expression Profiling) to analyze 392 DLBCL samples, looking for the CNV associated with the differential gene expression levels. Among the abnormal genomic regions identified by them, 32 turned out to be overlapped between at least two datasets. Based on the data, they defined 36 minimal common regions (MCR), several of which overlapped with peaks defined by GISTIC program, among which gains on 2p16.3–p14 (*REL*) and 9q34.11–q34.3 (*NOTCH1*) and losses on 1p13.2–p12 (*CD58*), 6p12.3–q27 (*TNFAIP3)*, 9p24.3–p21.1 (*JAK2*, *CDKN2A)*, 10q23.2–23.32 (*FAS*) and 19p13.3–p13.2 (*CD70*) can be mentioned [47].

As expected, SNP arrays have helped to define better the possible role of human genes in malignancies. For example, a locus in chromosomal location 15q15 encoding for p53-Binding protein 1 (*53BP1*), the protein product of which has a major role in DNA double strand break repair, was reported to be a subject of single copy loss in 9 out of 63 (14.5%) cases of DLBCL, as indicated by Takeyama *et al.* [44]. Interestingly, the same authors found a significant decrease in the gene expression level of 53BPI in the related tumor cases, indicating for the first time a possible role of this gene in human malignancies. Of note, this was the first report of such a role in human tumors [44]. Furthermore, although analyzing a limited number of DLBCL cases (*n* = 18), by combining SNP array technology and transcriptome profiling, Green *et al.* [40] found genetic lesions that significantly enriched for apoptosis and the mitogen activated protein kinase pathways. They were able to recognize two recurrent amplifications in DLBCL primary tumors, including 12p13.33 targeting *FOXM1* and 12q13.13, targeting *MAP3K12*. The authors argued the possible role of FOXM1 in non-hodgkin lymphomagenesis, demonstrating its possible association an increased MYC oncogenic signaling signature [40]. In another study on 242 DLBCL cases, Conde *et al.* [48] found a duplication for the chromosomal region 11q25 in 6.2% of cases. Interestingly, this region encodes for a long non-coding RNA (*LOC283177*), which further highlights the accumulating evidences for the role of this family of RNAs in human diseases.

Remarkably, some CNV patterns appeared significantly related with treatment response (when R-CHOP was considered as therapy) and overall survival [38,41,45,49]. Specifically, first of all, based on a study on 203 samples, the LMPP (Leukemia/Lymphoma Molecular Profiling Project) provided the genetic evidence that ABC- and GCB-DLBCL are distinct. In fact, they identified 272 recurrent chromosomal aberrations associated with gene expression alterations, 30 of which could efficiently separate the two DLBCL subtypes (*p* < 0.006) [45]. Among them, an amplicon on chromosome 19 was detected in 26% of ABC-DLBCLs but in only 3% of GCB-DLBCLs. A highly up-regulated gene in this amplicon was *SPIB*, which encodes an ETS family transcription factor and which was later on functionally related the pathogenesis of ABC-DLBCL. Similarly, Deletion of the *INK4a*/*ARF* tumor suppressor locus and trisomy 3 (leading to the over-expression of *FOXP1*) also occurred almost exclusively in ABC-DLBCLs and were associated with inferior outcome within this subtype. By contrast, in GCB-DLBCL, amplification of the oncogenic miR-17-92 microRNA cluster and deletion of the tumor suppressor *PTEN* were recurrent, but were not observed in the ABC-subtype [45].

Subsequently, Scandurra *et al.* [41] studied 166 primary samples and identified 20 recurrent genetic lesions that showed an impact on the clinical course. Among them, lesions with the strongest association with a worse outcome were deletions affecting the short arm of chromosome 8, including del(8p23.1) (*p* = 0.002), del(8p) (*p* = 0.01), and del(8p23.1–21.2) (*p* = 0.012). The loss of genomic material at 8p23.1 also appeared to be associated with additional aberrations, such as 17p- and 15q-. Overall, seven pathways appeared to be significantly enriched within the loci affected by CNV associated with survival: regulation of autophagy, natural killer cell-mediated cytotoxicity, antigen processing and presentation, Toll-like receptor signaling pathway, JAK-STAT signaling pathway, apoptosis and cytokine-cytokine receptor interaction. Finally, unsupervised clustering identified five DLBCL clusters with distinct genetic profiles, clinical characteristics (e.g., bone marrow involvement) and outcomes [41]. In another study performed by the GELA group, it was found that losses of *TP53* and *CDKN2A*, observed in 8% and 35% of 114 DLBCL patients analyzed, respectively, were significantly associated with a shorter survival after R-CHOP treatment, independently of the International Prognostic Index [49]. Analysis of the 9p21 genomic region indicated that transcripts encoding p14ARF and p16INK4A were both disrupted in most patients with *CDKN2A* deletion. These patients predominantly had an ABC-profile and showed a specific gene expression signature, characterized by deregulation of the RB/E2F pathway, activation of cellular metabolism, and decreased immune and inflammatory responses [49]. More recently, Monti *et al.* [38] studies 180 primary cases and showed that DLBCL cases showed either multiple complementary alterations of *TP53* and cell cycle components or largely lacked such lesions. DLBCLs with *TP53* and cell cycle pathway copy number abnormalities had decreased abundance of p53 transcriptional targets and increased expression of E2F target genes as well as increased Ki67 proliferation marker and poor clinical outcome [38].

Finally, some frequent regions showing LOH, including 11p11.2 have been identified in DLBCL cases. The latter which affects *PTPRJ*, was reported in 38% (16/42) of primary DLBCLs, indicating the LOH (and possible subsequent inactivation) of *PTPRJ* as a recurring event in NHLs [46].

### 3.2. Follicular Lymphoma

Follicular lymphoma (FL) is the second most common NHL subtype worldwide and possibly the most common in USA. Despite the clear differences in tumor biology, morphology, and aggressiveness when compared to DLBCL [50,51], there are some common molecular pathogenetic mechanisms among the two tumors, and more notably, by gaining new genomic aberrancies, at least 20% of FLs convert to DLBCL during time [40,52,53]. The most frequent genetic aberrancy in FL is a t(14;18)(q32;q21) translocation, which is observed in about 90% of cases and causes an over-expression of *BCL2*, an important antiapoptotic molecule [54]. However, *BCL2* translocation is not sufficient for FL development. Furthermore, a small minority of cases lack this translocation possibly representing a genetically different subset; in fact, by SNPs array analysis it was documented that BCL2-negative FL is characterized by a more simple genomic profile than the classical BCL2-positive ones [55].

In 2010, by using a multiplatform approach, including conventional cytogenetic techniques, BAC array comparative genomic hybridization, and Affymetrix 500K SNP arrays, Cheung *et al.* [56] studied a series of 50 FL cases for which normal matched DNA was available. This approach allowed to identify, in addition to the t(14;18), eight unique balanced translocations, including t(1;11;3), t(3;16)(q27;p13), t(2;4)(p16;q28) and t(2;4). The previously reported FL-associated copy number regions identified in their study were revealed including losses of 1p32-36, 6q, and 10q, and gains of 1q, 6p, 7, 12, 18, and X. The most frequent regions affected by CN-LOH turned out to be 1p36.33 (28% of cases), 6p21.3 (20%), 12q21.2–q24.33 (16%), and 16p13.3 (24%). Such regions were similarly affected in cases with more or less complex karyotype [56,57]. Of interest, the high resolution of the SNPs array allowed to identify 45 aberrant regions affecting one gene each, including *CDKN2A*, *CDKN2B*, *FHIT*, *KIT*, *PEX14*, and *PTPRD* (Table 2) [56].

**Table 2:** The most important recurrent genetic aberrations in Follicular Lymphoma (FL).

Location	Alteration	Candidate Genes	References
1p32-36	Loss	-	[56]
1p36	Loss	*TNFRSF14, PRDM16*	[58,59]
1p36.22	Gain	*PEX14*	[56]
1p36.33	Gain	*TNFRSF14*	[59]
1p36	CN-LOH	*TNFRSF14, PRDM16*	[56,57,58,60,61]
1q	Gain	-	[56]
2p16	Gain	*REL*, *BCL11A*	[59]
3p14	Loss, Gain	*FHIT*	[56]
3q27 *	Gain	*BCL6*	[59]
4q12	Loss, Gain	*KIT*	[56]
5p	Gain	-	[58,59]
6p	Gain	*CCND3*	[56,58,59]
6p	CN-LOH	-	[56,57,60,61,62]
6q	Loss	*TNFAIP3*	[56,58,59]
6q	CN-LOH	-	[60,61]
7p	Gain	*CARD11*, *RNF216*	[56,59]
8q24	Gain	*MYC*	[59]
9p21 *	Loss	*CDKN2A/2B*	[56,59]
9p	Loss	*PTPRD*	[56]
10q24	Loss	*LCOR*	[56,59]
10q	CN-LOH	-	[60,61]
11 *	Gain	-	[59]
11p11	LOH	*PTPRJ*	[46]
12q	Gain	*ARID2*, *HDAC7*, *RPAP3*	[56,58,59]
12q	CN-LOH	-	[56,57,60,61,62]
15q21 *	Loss	*B2M*	[59]
16p	CN-LOH	-	[56,57,60,61]
17p13	Loss	*TP53*	[59]
17q	Gain	-	[59]
18q21	Gain	*MALT1*, *BCL2*	[56,59]
21	Gain	-	[59]
X	Gain	-	[56,59]

* These aberrations occur in transformed follicular lymphoma (tFL).

Several other studies have pointed at the recurrence of CN-LOH in FL, and the regions influenced include 1p36, 6p, 6q, 10q, 12q and 16p [58,60,61,62], which in case of acquired UPD on 1p36 and 16p were correlated with shorter overall survival and poorer progression-free survival for the former and latter, respectively [61]. In another study, the recurrent CNVs in FL were reported to be overall associated with Wnt/b-catenin signaling and G1/S checkpoint regulation [63]. Furthermore, FL showed LOH of *PTPRJ* gene associated with LOH of 11p11.2 as it was observed in DLBCL [46]. In this regard, importantly, SNPs array karyotyping was recently used to dissect the genetic imbalances associated with FL transformation to DLBCL [59]. With this aim, Bouska *et al.* [59] studied 277 lymphoma samples (198 FL and 79 transformed FL/tFL) by both SNPs and gene expression microarrays. Common recurrent chromosomal abnormalities in FL included gains of 2, 5, 7, 6p, 8, 12, 17q, 18, 21, and X and losses on 6q and 17p. Many frequent small abnormalities, including losses of 1p36.33–p36.31, 6q23.3–q24.1, and 10q23.1–q25.1 and gains of 2p16.1–p15, 8q24.13–q24.3, and 12q12–q13.13 were also observed. Noteworthy, several candidate genes that may be affected were identified, including *TNFRSF14*, *PRDM16*, and the p53-family member *TP73* (Table 2)*.* Recurrent abnormalities more frequent in tFL samples included gains of 3q27.3–q28 and chromosome 11 and losses of 9p21.3 and 15q. Overall, abnormalities associated with disease transformation appeared to impair immune surveillance, activate the NFκB pathway, and deregulate p53 and B-cell transcription factors. A total of four abnormalities, namely gain of X or Xp and losses of 6q23.2–24.1 or 6q13–15, were found to be of clinical importance, *i.e.*, were significantly associated with an overall poor survival [59].

### 3.3. Mantle Cell Lymphoma

Mantle cell lymphoma (MCL) is a neoplasm of mature B-lymphocytes with characteristic t(11;14) (q13;q32) and subsequent *CCND1* (encoding for the cyclin D1) overexpression. Despite these unifying features, MCL represents a heterogeneous disorder, ranging from indolent MCL with benign clinical course to highly aggressive variants, sometimes characterized by peculiar blastoid or pleomorphic morphology [64,65]. As mentioned, the t(11;14)(q13;q32) with deregulated cyclin D1 expression is the primary genomic alteration in MCL. However, this aberration is not sufficient for lymphomagenesis [66,67], additional events being requested. Importantly, SNPs array karyotyping clearly indicated that the acquisition of a more complex karyotype is associated with a more aggressive clinical behavior. In particular, on the clinical point of view, the main diagnostic issue is to distinguish the indolent subtype *vs.* the classical (more aggressive) subtype. While indolent MCL usually shows stable genome without many secondary chromosomal abnormalities other than t(11;14)(q13;q32), classical MCL, and specially those characterized by blastoid and pleomorphic morphology, typically exhibit a complex karyotype with highly unstable genome [68,69]. Cytogenetic studies with comparative genomic hybridization microarray (CGH) have revealed frequent secondary gains and losses, involving genes important for genomic stability, proliferation, and apoptosis, such as *TP53*, *ATM*, *MYC*, *BMI1*, *CDK4* and *BCL2* [68,69,70] (Table 3). Among the large numbers of genes implicated in the lymphomagenesis of MCL, *MYC* appears to play an important role in transforming the initial B-cell clone harboring t(11;14)(q13;q32). MCL with both *CCND1* and *MYC* gene rearrangements is specified as “double hit” MCL [71]. In addition to *MYC*, other genes are also likely to be implicated in the aggressive behavior of lymphoma cells in double hit MCL. *CDKN2A* gene on chromosome 9 encodes two proteins by alternative splicing, INK4A and ARF, which are important in cell cycle control and stability of p53 respectively. Rubio-Moscardo *et al.* [72] showed that deletions of *INK4a* gene were associated with poorer prognosis together with blastoid morphology, increased number of genomic gains, and *TP53* gene deletion. Based on these findings, assessment of the *CDKN2A* gene locus in double hit MCL pathogenesis will be important in future studies as *CDKN2A* gene deletion might play a role in the pathogenesis of double hit MCL [71]. Finally, another high resolution SNPs array based study identified a duplication/amplification that occurred at 13q involving the oncogenic microRNA, miR17-92, the same previously observed to be deregulated in DLBCL (see above) (Table 3) [73].

**Table 3:** The most important recurrent genetic aberrations in Mantle cell lymphoma (MCL).

Location	Alteration	Candidate Genes	References
1p	Loss	-	[70]
3q	Gain	*SIAH2*, *PIK3CA*, *ACTL6A*, *YEATS2*, *RFC4*, *CENTB2*, *PAK2*	[68,70]
8p	Loss	*ESCO2*, *CLU*, *TNFRSF10D*, *ASAH1*	[70]
8q	Gain	*MYC*	[68,69,70]
9p	Loss	*CDKN2A* , *INK4a*	[68,70]
9q	Loss	*FANCC*, *XPA*, *RAD23*	[70]
10p	Gain	*BMI1*	[69]
11q	Loss	*ATM*, *CASP1*, *CASP4*, *BIRC2*	[68,69,70]
12q	Gain	*CDK4*	[69]
13q	Loss	*ERCC5*, *LIG4*	[68,70]
13q	Gain	*miR17-92*	[73]
15q	Gain	-	[70]
17p	Loss	*TP53*	[68,69,70]
18q	Gain	*BCL2*	[69]

### 3.4. Marginal Zone Lymphomas

Marginal zone B-cell lymphomas (MZLs) are currently divided into three distinct subtypes (extranodal MZLs of mucosa-associated lymphoid tissue (MALT) type, nodal MZLs, and splenic MZLs) according to the WHO Classification. However, the pathobiological differences as well as the relationship between the subtypes are still not well defined. In 2011, Rinaldi *et al.* [74] performed an extensive genomic analysis of DNA copy number changes in a large series of MZL cases, aiming to identify differences among subtypes. To do this, the Authors applied the Affymetrix Human Mapping 250K SNP array to 218 MZL samples (25 nodal, 57 MALT, 134 splenic, and two not better specified MZLs). First, it was found that MALT lymphoma presented significantly more frequently gains at 3p, 6p, 18p, and del(6q23) (*TNFAIP3*/*A20*). The latter finding, as A20 is a negative regulator of the NFκB pathway, was consistent with the activation of this pathway that is a frequent event in MALT lymphoma, more often due to chromosomal translocations. On the other hand, splenic MZLs was associated with del(7q31) and del(8p). Nodal MZLs lacked the splenic MZLs-related 7q losses but it did not show any statistically significant difference when compared with MALT lymphoma. Gains of 3q and 18q were common to all subtypes, confirming the existence of common pathogenetic pathways in the three variants (Table 4). From a clinical perspective, though del(17p), affecting *TP53*, did not determine a significantly worse outcome and del(8p) was only of borderline significance, the presence of both deletions (indeed often associated) had a highly significant negative impact on the outcome of splenic MZLs [74].

**Table 4:** The most important recurrent genetic aberrations in Marginal zone B-cell lymphomas (MZLs).

Malignancy	Location	Alteration	Candidate Genes	References
MZL (all 3 subtypes)	3q	Gain	*NFKBIZ*, *BCL6*	[74]
	18q	Gain	*BCL2*, *NFATC1*	[74]
MALT*	2p15	Gain	*REL*, *BCL11A*	[75]
	3p	Gain	*FOXP1*	[74]
	6p	Gain	*-*	[74]
	6q23	Loss	*TNFAIP3/A20*	[74]
	8p11	Gain-Loss	*ADAM3A*	[75]
	10q23	Gain	*PTEN*	[75]
	11q24	Gain	*ETS1*	[75]
	12p12	Gain	*KRAS*	[75]
	15q24	Gain-Loss	*SCAPER*	[75]
	18p	Gain	-	[74]
	20p13	Gain-Loss	*SIRPB1*	[75]
	20q13	Gain	*PTPN1*	[75]
	1p34	aUPD	*PRDX1*, *MUTYH*	[75]
	1p36.11-12	aUPD	*E2F2*, *ASAP3*	[75]
	1q43-q44	aUPD	*AKT3*	[75]
	2p23-24	aUPD	*TP53I3*	[75]
	6q21	aUPD	*TRAF3IP2*,*FYN*	[75]
	17q12	aUPD	*MED1*, *ERBB2*, *GRB7*, *IKZF3*	[75]
	17q23-24	aUPD	*PECAM1/CD31*	[75]
Splenic MZL	7q	Loss	*POT1*, *MIR29A*, *MIR29B*	[74]
	8p	Loss	-	[74]
	17p	Loss	*TP53*	[74]
Ocular adnexal MALT	6p	Gain	-	[76]
	6q	Loss	-	[76]
	9p	Loss	-	[76]
	21q	Gain	-	[76]
	6q	aUPD	-	[76]

*MALT: mucosa-associated lymphoid tissue.

More recently, another group focused on MALT lymphomas, aiming to identify by SNPs array karyotyping, possible genomic changes associated with disease progression [75]. Indeed, they included seven cases characterized by small cell morphology, eight composite lymphomas (*i.e.*, cases with small cell morphology and areas constituted by large cells) and 13 large cell variants using the Affymetrix Genome-Wide Human SNP Array 6.0 array. Consistent with the initial hypothesis, the Authors found an increase of genomic complexity with lymphoma progression from small to large cytology, and identified gains of well known oncogenes, including *REL*, *BCL11A*, *ETS1*, *PTPN1*, *PTEN* and *KRAS*, which were found exclusively in the large cell variants (Table 4). Copy numbers of *ADAM3A*, *SCAPER* and *SIRPB1* also varied among the three cytological subtypes, again indicating the presence of aberrations associated with progression from small to large cell lymphoma. Interestingly, the observation that the number of aberrations was slightly higher in the large cell part of composite lymphomas as compared to large cell lymphomas raised the hypothesis that large cells areas within composite lymphomas may represent a transition state of clonal selection during the progression from small cell to large cell variants. In line with this, when the cytologically different portions of two cases were analyzed in depth, an increase of genomic complexity from the small cell to the more blastoid part of the lymphoma was observed [75]. Interestingly, SNPs array analysis highlighted the frequency of acquired UPD in MZL, a previously uncovered phenomenon [74,75]. Among other loci, aUPD interested regions containing *PECAM1*/*CD31*, *PRDX1*, *E2F2*, *AKT3*, and *TRAF3IP2* (Table 4) [75]. Furthermore, a very recent analysis of 29 ocular adnexal MALT lymphomas, as reported by Takahashi *et al.* [76], revealed a higher occurrence rate of gains, as compared to losses and UPDs. Indeed, the authors reported gains including trisomy 3 in 31% of cases, trisomy 18 identified in 17% of samples, and 6p and 21q in 14% of tumors. On the other hand, losses of 6q and 9p were detected in 7% of all cases, along with UPD of 6q in 14% of cases.

### 3.5. Peripheral T Cell Lymphomas

Peripheral T cell lymphomas (PTCLs) are a heterogeneous group of cancers characterized by extremely variable morphology, phenotype, genetic and clinical features. The latter, however, mainly consist in very aggressive behavior and poor clinical outcome for most nodal tumors [77,78]. As the majority of PTCLs cannot be currently classified within a specific group based on morphology, phenotype, genetics and clinical features, they were included within the not otherwise specified group (PTCL/NOS) [79]. The latter, together with angioimmunoblastic T-cell lymphoma (AITL) and anaplastic large cell lymphomas (ALCL) ALK-positive and ALK-negative, represent the commonest nodal PTCL. Probably due to their rarity in Western countries, where they account for not more than 10% of all NHL, only relatively few genomic studies have been performed in this setting, specially based on SNPs arrays [80,81,82]. In 2008, Fujiwara *et al.* [80] used a GeneChip 50k SNP array to evaluate genetic perturbations in PTCL/NOS and AITL. They recognized some recurrent gains and losses, with chromosomal regions 8q24, 9p23 and 19q13 for the former and 9p21 for the latter (Table 5). Interestingly, the recurrent loss mapped to *CDKN2A* and *CDKN2B*, two important inhibitors of cyclin-dependent kinases with established role in human cancers. Subsequently, our group, by using the Affymetrix 250k GeneChip SNP array could achieve a higher resolution in defining the recurrent CNV and provided some additional details as far PTCL/NOS were concerned [81]. Indeed, we recognized a set of recurrent imbalances, including gains on chromosome 1, 2, 7, 8, 11, 17 and 21, and losses on chromosomes 1, 5, 6, 8, 9, 10, 13, 15, 16, 17 and X chromosome. Interestingly, genomic imbalances affected several regions containing members of nuclear factor-κ (NFκB) signaling. Particularly, gains of 2p15-16 were found and confirmed by FISH in three cases and were associated with breakpoints at the *REL* locus. Nuclear expression (*i.e.*, activation) of the encoded REL protein was then documented by immunohistochemistry. These findings were in line with what previously observed by gene expression profiling in PTCL/NOS and with the general notion that NFκB is activated in a fraction of cases with possible prognostic impact [83,84,85,86]. Further, recurrent genomic imbalances appeared to affect and genes involved in cell cycle control such as *RB1*, *TP53*, *CDK6* and *CDKN2A*-B (Table 5) [81].

**Table 5:** The most important recurrent genetic aberrations in Peripheral T cell lymphomas (PTCLs).

Location	Alteration	Candidate Genes	References
2p15	Gain	*REL*	[81]
6q21	Loss	*PRDM1*	[82]
7q21	Gain	*CDK6*	[81]
8q24	Gain	*EIF3H*	[80,81]
9p21	Loss	*CDKN2A*, *CDKN2B*, *MTAP*	[80,81]
9p23	Gain	-	[80]
10p11	Loss	*ZEB1*, *ARHGAP12*, *KIF5B*, *EPC1*, *CCDC7*	[81]
13q14	Loss	*RB1*	[81]
17p11	Loss	-	[81]
17p13	Loss	*TP53*	[81,82]
19q13	Gain	-	[80]

More recently, a high-density SNPs array was applied to a series of 64 ALCLs [82], the third most common nodal PTCL subtype. The authors found that the commonest lesions were losses at 17p13 and at 6q21, encompassing the *TP53* and *PRDM1* genes, respectively. The latter gene, encoding for BLIMP1, was inactivated by multiple mechanisms, more frequently, but not exclusively, in ALK-negative/ALCL. *In vitro* and *in vivo* experiments showed that that *PRDM1* is a tumor suppressor gene in ALCL models, likely acting as an anti-apoptotic agent. Losses of *TP53* and/or *PRDM1* were present in 52% of ALK-negative/ALCL, and in 29% of all ALCL cases. Of clinical relevance, the group of patients with ALK-negative/ALCL and bearing *PRDM1* and/or 17p loss presented an inferior overall survival [82].

## 4. Conclusions

With the advent of SNP arrays, a large number of genetic abnormalities have been described. This is mainly a result of its improved resolution compared to the more traditional methods like CGH and FISH. The newly discovered genetic alterations, such as CNVs and LOHs, have improved our understanding of the pathology of different human tumors, as well as provided us with new markers for diagnosis and therapy selection [11,12,13,14,15,16]. However, as any other technique, it has its own limitations; particularly, the lack of possibility for detecting inversions and balanced chromosomal translocations and the need, if optimal results are aimed, for normal matched DNA, has largely limited an extensive clinical application so far. However, a combination of SNP array along with other techniques such as FISH has often been accommodated [87,88]. Currently, NGS-based approaches are overcoming the limits of SNP arrays and cytogenetic technologies. SNP arrays, however, probably will continue to be used in diagnosis, as they offer more economic possibilities when compared to the current NGS options [88,89,90].
